# Delta Procalcitonin Is a Better Indicator of Infection Than Absolute Procalcitonin Values in Critically Ill Patients: A Prospective Observational Study

**DOI:** 10.1155/2016/3530752

**Published:** 2016-08-15

**Authors:** Domonkos Trásy, Krisztián Tánczos, Márton Németh, Péter Hankovszky, András Lovas, András Mikor, Edit Hajdú, Angelika Osztroluczki, János Fazakas, Zsolt Molnár

**Affiliations:** ^1^Faculty of Medicine, Department of Anaesthesiology and Intensive Therapy, University of Szeged, 6 Semmelweis Street, Szeged 6725, Hungary; ^2^Faculty of Medicine, Division of Infectious Diseases, First Department of Internal Medicine, University of Szeged, Szeged, Hungary; ^3^Faculty of Medicine, Department of Transplantation and Surgery, Semmelweis University, Budapest, Hungary

## Abstract

*Purpose*. To investigate whether absolute value of procalcitonin (PCT) or the change (delta-PCT) is better indicator of infection in intensive care patients.* Materials and Methods*.* Post hoc* analysis of a prospective observational study. Patients with suspected new-onset infection were included in whom PCT, C-reactive protein (CRP), temperature, and leukocyte (WBC) values were measured on inclusion (*t*_0_) and data were also available from the previous day (*t*_−1_). Based on clinical and microbiological data, patients were grouped* post hoc* into infection- (I-) and noninfection- (NI-) groups.* Results*. Of the 114 patients, 85 (75%) had proven infection. PCT levels were similar at *t*_−1_: I-group (median [interquartile range]): 1.04 [0.40–3.57] versus NI-group: 0.53 [0.16–1.68], *p* = 0.444. By *t*_0_ PCT levels were significantly higher in the I-group: 4.62 [1.91–12.62] versus 1.12 [0.30–1.66], *p* = 0.018. The area under the curve to predict infection for absolute values of PCT was 0.64 [95% CI = 0.52–0.76], *p* = 0.022; for percentage change: 0.77 [0.66–0.87], *p* < 0.001; and for delta-PCT: 0.85 [0.78–0.92], *p* < 0.001. The optimal cut-off value for delta-PCT to indicate infection was 0.76 ng/mL (sensitivity 80 [70–88]%, specificity 86 [68-96]%). Neither absolute values nor changes in CRP, temperature, or WBC could predict infection.* Conclusions*. Our results suggest that delta-PCT values are superior to absolute values in indicating infection in intensive care patients. This trial is registered with ClinicalTrials.gov identifier: NCT02311816.

## 1. Introduction

Treatment of severe sepsis and septic shock remains a major challenge in the critically ill, and it is still one of the leading causes of death worldwide [1]. Despite increased awareness of the importance of early resuscitation, mortality in North America and Europe ranges between 28 and 41% [2]. Based on a consensus agreement sepsis is defined as infection in the presence of systemic inflammatory response syndrome (SIRS) [3]. However, the signs of SIRS are nonspecific and can often be seen in several (none septic) critically ill conditions. Fever, tachycardia, or leukocytosis on their own has low sensitivity and specificity [4, 5]. Detailed microbiological results are often only available after 24 hours or later, and negative results do not necessarily rule out infection. Nevertheless, early diagnosis of infection in critically ill patients is of utmost importance, and delay in starting appropriate antibiotic therapy may lead to lethal events [6]. However, giving antibiotics unnecessarily to every acutely ill patient is an unacceptable practice for several reasons [7]. Therefore, fast reacting biomarkers of infection have been used for almost 50 years to help the clinician, of which C-reactive protein (CRP) and procalcitonin (PCT) are the most often used and studied [8].

Procalcitonin is a fast reacting biomarker with a half-life of around 24 hours [9]. Its sensitivity and specificity for bacterial infection seem to be superior compared to CRP [10, 11]. However, it must be considered that the same absolute values of PCT cannot be used in all circumstances. It has been reported that PCT levels are higher in surgical compared to medical patients [12], and elevated PCT can also be present without infection, in conditions such as trauma [13] and surgery [14] or after cardiac arrest [15]. There is some evidence that evaluating PCT kinetics may be superior to absolute values [12, 16].

In this study, our aim was to investigate whether the absolute value of PCT measured in critically ill patients on the day when infection was suspected, or the change in PCT (delta-PCT) from the day before to the day when infection was suspected, was a better indicator of infection.

## 2. Methods

### 2.1. Patient Selection

This prospective observational study was part of the Early Procalcitonin Kinetics (EProK) study, which was undertaken between October 2012 and October 2013 and approved by the Regional and Institutional Human Medical Biological Research Ethics Committee, University of Szeged, Hungary (WHO-3005; 19.04.2012, Chairperson Professor T. Wittmann). A detailed description of the EProK study and the final results are published elsewhere [17]. The investigation was performed at the University of Szeged (Szeged, Hungary), Albert Szent-Györgyi Health Center in four tertiary intensive care units. The study was registered at ClinicalTrials.gov with the registration number: NCT02311816. Written informed consent was obtained from all subjects or from their relatives.

#### 2.1.1. Inclusion Criteria

In the EProK study all patients over 18 years with suspected infection on admission or during their stay on the intensive care unit were screened for eligibility. Patients were enrolled, when the attending intensive care specialist suspected infection, based on (1) suspected source which could be identified, (2) new onset organ dysfunction, and (3) body temperature, PCT, CRP, and the decision to start empirical antibiotic therapy. Once the original EProK study was completed, in a* post hoc* analysis those patients in whom PCT and CRP values were available from the previous day (*t*_−1_) were included in the current analysis.

#### 2.1.2. Exclusion Criteria

Exclusion criteria included patients younger than 18 years, who had received antibiotic therapy in the previous 48 hours, and those who received acute renal replacement therapy 24 hours before enrollment. Patients were also excluded following cardiopulmonary resuscitation and with end stage diseases with a “do not resuscitate” order. Immunocompromised patients (human immunodeficiency virus infection, bone marrow transplantation, malignant haematological disorders, and chemotherapy) were also excluded.

### 2.2. Subgroups and Definitions

Diagnosis of infection was based on a* post hoc* analysis of mainly microbiological results but also clinical parameters and biochemical results which were evaluated by two experts (infectologist, EH, and an intensivist, FJ) blinded for the PCT data apart from the first PCT measurement (*t*_0_, see below). The experts also took into consideration the recommendations of international guidelines [18, 19]. Based on these results, patients were grouped into “infection-” (I-) and “noninfection-” (NI-) groups.

For subgroup analysis, patients were divided into “medical” and “surgical” groups. The medical-group represented patients who had had no surgical intervention before and during the study period and for source control did not require surgery. In the surgical-group infection either was related to an operation or required surgery for source control [12].

### 2.3. Protocol and Data Collection

Whenever infection was suspected by the attending physician, the signs of infection and the suspected source were recorded, which included high/low body temperature (<36°C; >38°C), high/low white blood cell count (<4,000; >12,000 million/mL), acute worsening of the clinical picture (hemodynamic instability, worsening PaO_2_/FiO_2_ ratio, and deterioration in mental status or any other clinical sign indicating infection). Microbiological specimens were collected from all suspected sources immediately before the administration of the first dose of antibiotics (*t*_0_).

#### 2.3.1. Data Collection

After enrollment, demographic data, signs of infection, the suspected source of infection, and corresponding microbiological samples were registered. The length of intensive care unit and hospital stay, 28 days, and the overall mortality were also documented.

#### 2.3.2. Procalcitonin Measurement

**Figure 1:**
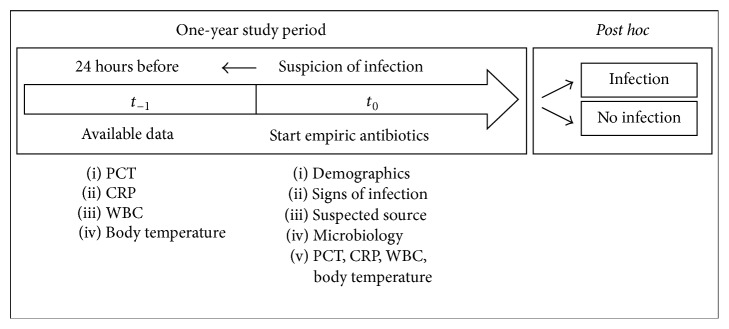
Flow chart.

It is common practice in our ICU to measure PCT daily in critically ill patients. Procalcitonin levels were documented from the previous day of enrollment (*t*_−1_) and immediately before the initiation of ABs (*t*_0_). Core temperature, C-reactive protein (CRP), and white blood cell count (WBC) were also recorded with every PCT measure. The flow chart of the data collection is summarised in Figure 1.

Serum PCT levels were measured with Cobas 6000 analyzer (Hitachi High-Technologies Corporation, Tokyo, Japan). Analyzer reagents (Elecsys® B·R·A·H·M·S PCT assay) were developed in collaboration with B·R·A·H·M·S corporation (Hennigsdorf, Germany) and Roche Diagnostics (Mannheim, Germany). Procalcitonin was determined by electrochemiluminescence immunoassay (ECLIA) serum on the automated Roche Elecsys and Cobas immunoassay analyzers.

#### 2.3.3. Microbiological Staining and Antibiograms

Microbiological tests were performed and sent at *t*_0_, before the first antibiotic dose was administered and if needed they were repeated on the following days, to identify infection.

### 2.4. Statistical Analysis

Data were analyzed using IBM SPSS Statistics Version 20 (Armonk, NY, USA) and Systat Software Inc. SigmaPlot 12.5 (London, UK) software. For continuous data, the Shapiro-Wilk tests were performed to assess normal distribution. Demographic data were analyzed between groups with Student's *t*-test or nonparametric data with the Mann-Whitney *U* test as appropriate. Categorical data were compared using *χ* ^2^ tests. Biomarkers were analyzed by using Two-Way Repeated Measures Analysis of Variances (All Pairwise Multiple Comparison Procedures: Holm-Sidak method). Logistic regression, receiver operating characteristic (ROC) curve, and the respective areas under the curves (AUC) were calculated for PCT, CRP, body temperature, and white blood cell count levels. The best cut-off values were determined using the Youden index (*J* = max⁡[Sens + Spec − 1]). The test parameters (sensitivity, specificity, positive, and negative predictive values) were compared by their 95% confidence intervals. Logistic regression analysis was used to determine the best combination of parameters and cut-offs for predicting infection. The level of *p* < 0.05 was defined as statistically significant. Data are given as mean ± standard deviation or median (interquartile range) as appropriate.

The “delta” was considered as the changes in the absolute values (subtracting *t*_−1_ from *t*_0_); the percentage values were calculated as [(*t*_0_/*t*_−1_) × 100 − 100].

## 3. Results

Over the one-year study period all ICU patients were screened for eligibility and 209 patients were recruited into the EProK study. Out of the 209 patients in the current* post hoc* analysis we include 114 cases where PCT values were available from the previous day. Demography and outcomes characteristics for the entire cohort are summarised in Table 1. Out of the 114 patients, 85 (75%) patients were identified as having proven infection and in 29 (25%) patients the presence of infection was highly unlikely. Disease severity scores and outcomes were similar in the two groups, but the NI-group required less organ support.

The clinical and laboratory signs of infection on which the clinicians suspected infection at the time of inclusion (*t*_0_) are summarised in Table 2. Although all indices were higher in the I-group, but only the altered level of consciousness, hemodynamic instability, and the PCT was significantly different between the two groups.

Regarding the suspected source of infection, generally there was nonsignificant difference between the groups, but significantly more patients were suspected of having abdominal related infection in the NI-group. Detailed data on the isolated pathogens and their sources are summarised in the Supplemental Digital Content Tables S5–7 (see Supplementary Material available online at http://dx.doi.org/10.1155/2016/3530752).

### 3.1. PCT, CRP, WBC, and Temperature Values at *t*_−1_ and *t*_0_

#### 3.1.1. Total Sample

**Figure 2:**
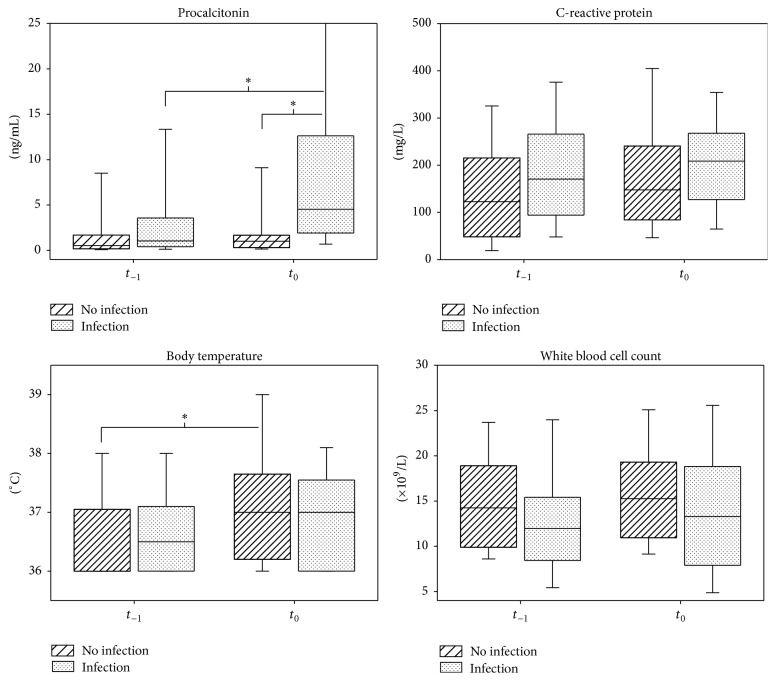
PCT, CRP, body temperature, and WBC count absolute values in the total cohort. Boxplots present median (interquartile range) 10th and 90th percentile. *∗* indicates *p* < 0.05.

Measurement results at *t*_−1_ and *t*_0_ in the I- and NI-groups are shown in Figure 2. PCT absolute values were similar at *t*_−1_, but by *t*_0_ in the I-group levels were significantly higher compared to the NI-group and there was also a significant increase from *t*_−1_, while there was no such change in the NI-group. There was no significant difference in CRP and WBC count between the two groups nor could we find significant changes from *t*_−1_ to *t*_0_. There was no difference between the groups for body temperature but there was a statistically significant increase in the NI-group by *t*_0_. It is of note that body temperature remained <38°C in almost all patients.

#### 3.1.2. Medical and Surgical Patients

Measurement results in medical (*n* = 80) and surgical (*n* = 34) patients are summarised in Table 3. In the surgical subgroup PCT absolute values were significantly higher than in the medical cohort, but the pattern of change was similar. In the NI-group there was a slight, but statistically significant increase in medical patients from *t*_−1_ to *t*_0_, while there was no significant change in surgical patients, where levels actually decreased slightly. However, in the I-group there was an almost 3-fold increase in the PCT levels.

Regarding the CRP, body temperature, and WBC count, there was no significant changes over time and no differences between medical and surgical patients.

### 3.2. Predictive Value for Indicating Infection

**Figure 3:**
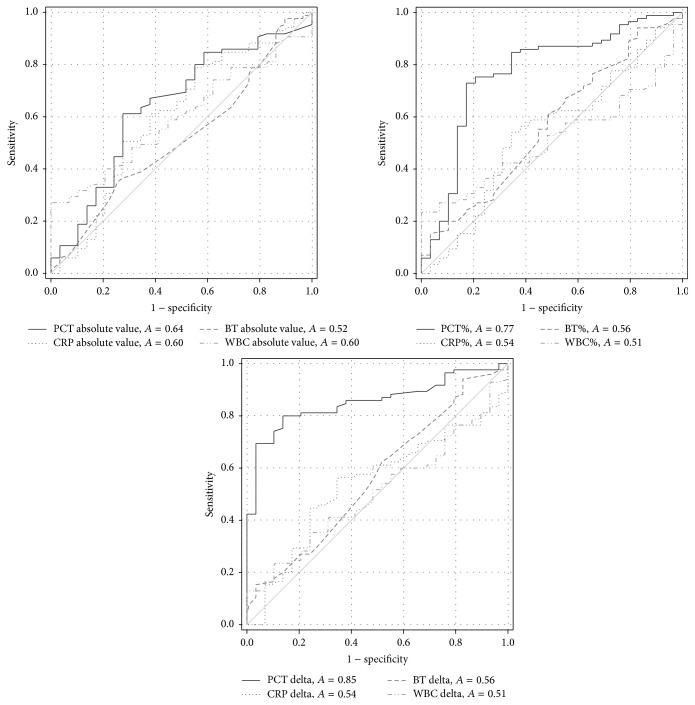
The predictive value of the absolute values, percentage, and delta changes of PCT, CRP, temperature, and WBC count for infection in the total cohort.

The predictive value for infection for the absolute values of PCT, CRP, temperature, and WBC count can be seen in Figure 3 and is summarised in Table 4. Only PCT had a significant predictive value, but with a poor AUC (Figure 3). However, regarding the percentage and delta changes CRP, temperature and WBC counts diagnostic value did not change, while PCT's AUC for both percentage and delta changes had a significantly better performance for predicting infection. Similar patterns were observed in the medical and surgical subgroups (Table 4).

### 3.3. Best Cut-Off Value

The best cut-off values were defined for PCT only as there was no significant predictive value for the other parameters, as determined by the Youden index. For the PCT absolute value it was 0.84 ng/mL with a sensitivity of 61% (95% CI: 50–72) and specificity 72% (53–87) to indicate infection in the ICU. Regarding the percentage change a PCT increase of >88% from *t*_−1_ to *t*_0_ had a sensitivity of 75% (65–84) and specificity of 79% (60–92) and a PCT delta change of >0.76 ng/mL had a sensitivity of 80% (70–88) and specificity of 86% (68–96) to indicate infection.

Data were also analyzed using the logistic regression model for finding the best combination of these four parameters together to predict infection in the ICU. However, none of the combinations tested improved the performance for predicting infection (data not shown).

## 4. Discussion

The main finding of this observational study was an increase in PCT levels from the day before (*t*_−1_) to the day when infection was suspected (*t*_0_) predicted infection, while in patients with no proven infection PCT remained unchanged. Furthermore, regarding the conventional indicators of infection such as WBC, body temperature, and CRP, neither the absolute values nor their change from *t*_−1_ to *t*_0_ could predict infection.

Diagnosing infection in the critically ill is challenging. Appropriate decision making has paramount importance as any delay in adequate antibiotic treatment of sepsis and septic shock evokes worsening morbidity and mortality results [6, 20]. On the other hand unnecessary antibiotic administration in patients without infection has led to the emergence of multidrug-resistant bacteria [21, 22], complications related to the side effects of the antibiotics themselves and an increased burden of healthcare expenses [23]. Despite its importance, there is no gold standard for diagnosing/proving infection in the critical care setting.

In our study 75% of patients had proven infection. This complex* post hoc* analysis of all results is fundamentally different from “labelling” patients as septic, based solely on the Surviving Sepsis Guideline criteria at the time of initial assessment as seen in several studies [24, 25]. Although our method also has some uncertainties, it provides a more robust approach utilising all data, clinical, biochemical, and microbiology alike, to aid in the diagnosis of patients with bacterial infection. However, it is also important to acknowledge that there is no gold standard to diagnose infection; therefore despite all our efforts, some patients in the NI-group may have had culture negative infection.

In our investigation it was found that conventional indicators of infection such as body temperature and white cell count had less value in diagnosing infection. Levels of WBC count remained elevated on both days and there was no significant change over time. This phenomenon can be explained by the nonspecific activation of the immune cascade as often seen in ICU patients [26]. Although there was a statistically significant increase in body temperature in the NI-group, levels largely remained below 38°C in almost all patients. These results are in accordance with recent findings that increased temperature alone does not predict infection [27].

Although microbiology remains the gold standard for confirming pathogens, results only come back at least 24–48 hours after sampling. Furthermore, in several cases results remained negative, despite obvious signs of infection. In order to help the diagnostic process several novel biomarkers of infection have been developed [8]. However, all biomarkers share the same limitations that “one size will not fit all,” due to the complex pathomechanism and the heterogeneity of patients.

The two most commonly used markers in infection/sepsis diagnostics are PCT and CRP [8]. Procalcitonin is detectable in the serum a few (2–4) hours after the onset of bacterial infection. It reaches its peak within 24 hours and then starts to decline with adequate treatment by around a 50% daily decrease according to its half-life [9]. In contrast, CRP has a delayed response. It reaches its maximum value usually after 48 hours of an insult and in general it lags behind the actual events of the inflammatory and clinical process. Furthermore, CRP levels are generally elevated in most ICU patients regardless of the aetiology. In our study neither the absolute values of CRP nor its delta changes were able to indicate new onset infection. Patients had elevated CRP values with a median of almost 200 mmol/L for the whole cohort, which makes interpretation very difficult. Furthermore the kinetics did not show any significant change over time. Therefore, our results question the place of CRP measurements for diagnosing infection on the ICU.

The most important finding of the current study was to show the superiority of PCT kinetics over the absolute values to indicate new onset infection in the ICU. However, this requires at least daily measurements of PCT, which has been common practice in our ICU in critically ill patients in whom infection cannot be excluded. Our current findings are in accordance with those reported by Tsangaris et al. [16]. They also measured PCT daily and observed a twofold increase of PCT levels from the day before to the day when there was a sudden onset of fever in patients with proven infection, but no change in PCT was found in patients without infection. They concluded that, in patients treated chronically in the ICU, PCT values on the day of fever onset must be compared to values measured the previous day in order to define whether this rise in temperature was due to infection or not. An important difference between their and our study is that in our patients body temperature merely reached 38°C; in fact most of these patients were apyrexial, despite 75% having proven infection. Therefore, we recommend to evaluate PCT kinetics not only in the onset of fever, but whenever infection is suspected on the ICU. Based on the current results, the best cut-off values were also determined for change in PCT, which were >88% and >0.76 ng/mL delta change from *t*_−1_ to *t*_0_. The reasons why a given absolute value of any biomarker, not just PCT, may be of limited value as compared to its changes can be explained by the pathomechanism of systemic inflammation. It was a very important discovery that after trauma, burns, ischemia-reperfusion, pancreatitis, major surgery, and so forth, the same or similar molecules are released predominantly from the mitochondria, as after an infectious insult. Based on aetiology these are called “damage-associated molecular patterns” (DAMP), or “pathogen associated molecular patterns” (PAMP). Once similar mediators/proteins are released they act on the same receptors of monocytes inflicting a similar inflammatory response, including PCT release and subsequent organ dysfunction [28, 29].

Indeed, PCT levels were found to be severalfold higher in surgical compared to medical patients in septic shock despite the similar clinical manifestation and severity of the clinical picture [12]. This explains why PCT levels were elevated in our surgical patient population without proven infection, with median values of around 3.5 (NI-group) and 3.8 (I-group) ng/mL at *t*_−1_. The corresponding PCT values in medical patients were substantially lower (0.26 and 0.89 ng/mL, resp.). Although levels were higher in the I-group at *t*_−1_, this difference did not reach statistical significance while there was a severalfold increase in the I-group in both medical and surgical patients with no change in kinetics in the NI-groups.

In two large recent multicenter trials the authors could not show any benefit from a PCT-based approach in antibiotic management in the ICU [30, 31]. However, in both studies the threshold for intervention was a PCT of >1 ng/mL. As 40% of the patients in both trials were surgical, in whom this threshold for intervention may be too low, one cannot exclude that these patients may have had received antibiotics unnecessarily. This overuse of antibiotics may be one of the reasons for the worse outcome in the PCT-guided group in both studies. Our study provides further evidence that changes or kinetics of PCT may be superior to absolute values.

The current study has several limitations. Firstly, one may argue that there was a selection bias; in other words, physicians suspected infection more often when they observed a PCT increase in a patient. Although this cannot be excluded completely, at the time when the study was performed, PCT collection was not the routine practice within the department, and delta-PCT was not included among the criteria of inclusion either. The whole idea of retrieving PCT data from the day before came after we analyzed the original EProK database. Secondly, despite all our efforts of allocating patients into the I- and NI-groups, this took place in a* post hoc* fashion. The available clinical results were analyzed in a blinded manner for delta-PCT (apart from PCT values at *t*_0_) and thoroughly by our experts; however, one cannot exclude the possibility of inappropriate judgment during the decision making. The lack of gold standard for diagnosing infection is aggravated by this obscurity when configuring groups. Furthermore, the sample size was generally small, especially to be able to draw firm conclusions regarding the medical, surgical subgroups, although the trend in our results was certainly promising. Finally, it remains uncertain why PCT values were measured on the previous day before starting empiric antibiotic therapy in more than 50% of the 209 patients of the EProK study. Therefore, some selection bias cannot be excluded. The median day of inclusion into the study from ICU admission was 1 day, indicating that 50% of patients had PCT measurements on the ward/Accident and Emergency Unit, before admission. However, this may also reinforce the importance of measuring PCT values consecutively.

## 5. Conclusion

The main finding of this observational study was that an increase in PCT levels from the day before (*t*_−1_) to the day when infection was suspected (*t*_0_) predicted infection, while in patients with no proven infection PCT remained unchanged. Based on the data presented a single PCT measurement may not be adequate to differentiate between an infectious and noninfectious inflammatory response. This means that the kinetics of procalcitonin values based on daily measurements are superior to absolute values in diagnosing infection on the ICU and absolute values of procalcitonin may be of limited use. Both absolute values and kinetics of C-reactive protein are poor indicators of infection; furthermore, conventional indicators of infection such as white cell count and body temperature have limited use for predicting infection in the ICU. The clinical implication of these results is that daily PCT measurements in patients at high risk of infection allow the opportunity to evaluate PCT kinetics, which may improve diagnostic accuracy and rationalise antibiotic therapy on the ICU and improve outcome.

## Supplementary Material

Supplemental Digital Content Tables S5-7: As a supplemental digital material a detailed summary of the results of the microbiological stainings can be seen. Table S5 shows the suspected sources of infection based on the clinical picture, laboratory tests and microbiological results. In the Table S6 the sources of positive and negative microbiological samplings sent for staining can be seen in the infection group. And in Table S7 the cultured pathogens and their source of sampling are collected.

## Figures and Tables

**Table 1:** Demographics, organ support, and outcome in the entire cohort.

	Total	NI-group	I-group	*p* value
Age (years)	65 (22.5)	67 (25.5)	65 (22)	0.772
Gender (M/F)	69/45	15/14	54/31	0.261
Body height (cm)	170 (12)	167 (19)	170 (11)	0.766
Body weight (kg)	73 (25)	80 (25)	70 (20)	0.345
SAPS II points	62.2 ± 20.5	62.7 ± 25.5	66.1 ± 18.6	0.513
SAPS II PM (%)	77.2 (52.1)	64.0 (75.9)	78.5 (42.1)	0.437
ICU days before enrollment	1 (3)	1 (3)	1 (3)	0.669
Mechanical ventilation	80 (70.2%)	12 (41.4%)	68 (80.0%)	<0.001
Vasopressor therapy	69 (60.5%)	13 (44.8%)	56 (65.9%)	0.045
ICU LOS (day)	9 (12)	8 (8)	9 (12)	0.089
ICU survival	84 (73.7%)	24 (82.8%)	60 (70.6%)	0.199
Hospital LOS (day)	17 (20)	14 (17)	19 (22)	0.050
Hospital survival	67 (58.8%)	20 (68.9%)	47 (55.3%)	0.197
28-day survival	64 (56.1%)	19 (65.5%)	45 (52.9%)	0.239

Data are given as mean ± standard deviation or median (interquartile range) as appropriate. M: male; F: female; SAPS: simplified acute physiology score; PM: predicted mortality; ICU: intensive care unit; LOS: length of stay; mechanical ventilation and vasopressor therapy represent data at the day of enrollment.

**Table 2:** Clinical signs and suspected source of infection at enrollment (*t*_0_).

	Total *n* = 114	NI-group *n* = 29	I-group *n* = 85	*p* value
Fever (<36°C; >38°C)	55 (48.2%)	13 (44.8%)	42 (49.4%)	0.670
WBC (>12 or <4 × 10^9^/L)	82 (71.9%)	22 (75.9%)	60 (70.6%)	0.585
Impaired gas exchange	82 (71.9%)	18 (62.1%)	64 (75.3%)	0.171
Impaired consciousness	59 (51.8%)	9 (31.0%)	50 (58.8%)	0.010
Hemodynamic instability	74 (64.9%)	13 (44.8%)	61 (71.8%)	0.009
PCT (ng/mL)	3.37 (9.22)	1.12 (1.36)	4.62 (10.72)	0.018
CRP (mg/L)	182.75 (158.5)	147.60 (156.50)	208.80 (140.60)	0.301

Respiratory	72 (63.2%)	17 (58.6%)	55 (64.7%)	0.557
Soft tissue	13 (11.4%)	2 (6.9%)	11 (12.9%)	0.377
Abdominal	14 (12.3%)	7 (24.1%)	7 (8.2%)	0.024
Urinary tract	5 (4.4%)	0	5 (5.9%)	0.182
Bloodstream	6 (5.3%)	2 (6.9%)	4 (4.7%)	0.648
Central nervous system	4 (3.5%)	1 (3.4%)	3 (3.5%)	0.984

WBC: white blood cell count, PCT: procalcitonin, and CRP: C-reactive protein. The PCT and CRP values are presented as median (interquartile range).

**Table 3:** PCT, CRP, body temperature, and white blood cell count in medical and surgical patients with and without infection.

		NI-group		I-group	
		*t*_−1_	*t*_0_	*t*_−1_	*t*_0_
Medical	PCT (ng/mL)	0.26 (0.57)	0.54 (1.16)^*∗*^	0.89 (1.52)	3.17 (5.9)^*∗*#^
	CRP (mg/L)	136.7 (159.1)	141 (125.9)	150 (184.3)	164.2 (145.3)
	BT (°C)	36 (1.02)	37 (0.82)^*∗*^	36.9 (1.23)	37 (1.6)
	WBC (×10^9^/L)	14.32 (8.9)	15.4 (8.64)	12.06 (6.36)	13.76 (10.16)

Surgical	PCT (ng/mL)	3.5 (9.91)	2.89 (9.33)	3.83 (22.55)	14.9 (58.06)^*∗*#^
	CRP (mg/L)	95 (342.5)	163 (327.4)	199.5 (130.1)	243.2 (112.7)
	BT (°C)	36.5 (2)	36.5 (2.4)	36 (1)	36.9 (1.1)
	WBC (×10^9^/L)	8.99 (7.37)	14.56 (9.65)	11.9 (10.06)	10.91 (9.9)

Data are presented as median (interquartile range). PCT: procalcitonin, CRP: C-reactive protein, BT: body temperature, and WBC: white blood cell count; ^*∗*^ *p* < 0.05 within groups and ^#^ *p* < 0.05 between groups.

**Table 4:** The predictive value of the absolute values, percentage, and delta changes of PCT, CRP, temperature, and WBC count for infection in the total cohort.

		Absolute value			Percentage changes			Absolute value changes		
		AUC	95% CI	*p* value	AUC	95% CI	*p* value	AUC	95% CI	*p* value
Total	PCT	0.64	0.52–0.76	0.022	0.77	0.66–0.87	<0.001	0.85	0.78–0.92	<0.001
	CRP	0.60	0.47–0.72	0.103	0.54	0.41–0.66	0.530	0.54	0.42–0.65	0.536
	BT	0.52	0.39–0.63	0.804	0.56	0.44–0.68	0.300	0.56	0.44–0.68	0.322
	WBC	0.60	0.48–0.70	0.125	0.51	0.40–0.61	0.852	0.51	0.39–0.61	0.924

Medical	PCT	0.67	0.54–0.80	0.016	0.76	0.63–0.88	<0.001	0.83	0.73–0.92	<0.001
	CRP	0.58	0.44–0.72	0.248	0.57	0.42–0.70	0.359	0.57	0.44–0.70	0.306
	BT	0.51	0.37–0.64	0.858	0.64	0.50–0.77	0.055	0.64	0.49–0.77	0.060
	WBC	0.57	0.44–0.70	0.329	0.56	0.43–0.68	0.441	0.57	0.43–0.69	0.365

Surgical	PCT	0.78	0.58–0.97	0.025	0.80	0.59–1.00	0.014	0.94	0.85–1.00	<0.001
	CRP	0.56	0.23–0.87	0.654	0.56	0.29–0.81	0.654	0.54	0.31–0.76	0.749
	BT	0.52	0.22–0.80	0.898	0.63	0.39–0.85	0.306	0.63	0.39–0.86	0.296
	WBC	0.63	0.44–0.82	0.277	0.67	0.47–0.86	0.166	0.71	0.49–0.90	0.108

AUC: area under the ROC curve, CI: confidence interval, PCT: procalcitonin, CRP: C-reactive protein, BT: body temperature, and WBC: white blood cell count.
