# Supplementary material for: Delta Procalcitonin Is a Better Indicator of Infection Than Absolute Procalcitonin Values in Critically Ill Patients: A Prospective Observational Study
Source: J Immunol Res. 2016 Aug 15;2016:3530752. doi: 10.1155/2016/3530752 (PMC5002471; doi:10.1155/2016/3530752)
Supplement: Supplementary file 1 — Supplemental Digital Content Tables S5-7: As a supplemental digital material a detailed summary of the results of the microbiological stainings can be seen. Table S5 shows the suspected sources of infection based on the clinical picture, laboratory tests and microbiological results. In the Table S6 the sources of positive and negative microbiological samplings sent for staining can be seen in the infection group. And in Table S7 the cultured pathogens and their source of sampling are collected. [file 3530752.f1.pdf]

Supplemental Digital Content

Table S5 Source of infection

Source	n	%
Abdominal	11	12,5
Central nervous system	5	5,68
Genitourinary tract	5	5,68
Intravascular catheter	5	5,68
Lung	54	61,36
Soft tissue	6	6,82
Unknown	2	2,27
Total	88	100

Table S6 Samples from the Infection group

Sample	Total	Positive	Negative
BAL	5	4	1
Blood culture	117	72	45
Catheter tip	5	4	1
CSF	16	16	0
Intra-abdominal	4	3	1
Nose	4	1	3
Permanent urinary catheter	18	4	14
Pleural fluid	3	3	0
Skin	2	0	2
Throat	1	0	1
Tracheal aspiration	68	58	10
Urine	16	14	2
Wound	22	20	2
Total	281	199	82

BAL - bronchoalveolar lavage, CSF – cerebrospinal fluid

Table S7 Pathogens from the infection group

**Pathogenes**	**Sample**	**n**
Acinetobacter baumannii	tracheal aspiration	2
Acinetobacter haemolyticus	blood culture	1
Acinetobacter spp.	wound	1
Arcanobacterium bernardiae	intra-abdominal	1
Bacillus cereus	CSF	1
Bacillus spp.	blood culture	2
Candida albicans	blood culture	1
	intra-abdominal	1
	tracheal aspiration	7
Candida non-albicans	blood culture	1
	tracheal aspiration	8
Clostridium perfringens	BAL	1
Clostridium spp.	wound	1
Comamonas testosteroni	blood culture	1
Eggerthella lenta	intra-abdominal	1
Enterobacter aerogenes	blood culture	3
	tracheal aspiration	2
Enterobacter cloacae	blood culture	1
	catheter tip	1
	intra-abdominal	1
	tracheal aspiration	5
	wound	1
Enterococcus faecalis	blood culture	6
	intra-abdominal	2
	urine	2
	wound	2
Enterococcus faecium	blood culture	3
	intra-abdominal	1
	PUC	1
Escherichia coli	blood culture	4
	intra-abdominal	3
	PUC	3
	tracheal aspiration	6
	urine	7
Filamentous fungi	nose	1
Finegoldia magna	wound	1
Fusobacterium spp.	pleural fluid	1
Gemella morbillorum	wound	1
Geotrichum capitatum	blood culture	1
	urine	1

Gram + not identified coccus	blood culture	2
Haemofilus influenzae	blood culture	3
	tracheal aspiration	4
Klebsiella oxytoca	blood culture	1
	tracheal aspiration	2
	wound	1
Klebsiella pneumoniae	BAL	1
	blood culture	2
	intra-abdominal	1
	CSF	1
	tracheal aspiration	1
	wound	2
Kocuria rhizophila	blood culture	1
Lactobacillus spp.	wound	1
Morganellamorganii	tracheal aspiration	1
	urine	1
	wound	1
MRSA	blood culture	4
Neisseria meningitidis	tracheal aspiration	1
Parvimonas micra	pleural fluid	1
	wound	1
Peptostreptococcus anaerobius	wound	1
Prevotella melaninogenica	wound	1
Propionibacterium acnes	BAL	1
Proteus mirabilis	intra-abdominal	1
	wound	2
Pseudomonas aeruginosa	blood culture	2
	catheter tip	1
	intra-abdominal	1
	CSF	1
	tracheal aspiration	4
	urine	3
	wound	1
Raoultella ornithinolytica	tracheal aspiration	1
Serratia marcescens	tracheal aspiration	2
Staphylococcus aureus	BAL	1
	blood culture	6
	catheter tip	1
	tracheal aspiration	8
	wound	2
Staphylococcus epidermidis	blood culture	18
Staphylococcus haemolyticus	intra-abdominal	1

Staphylococcus hominis	blood culture	4
	cathete rtip	1
	CSF	1
Stenotrophomonas maltophilia	blood culture	1
	tracheal aspiration	1
Streptococcus agalactiae	tracheal aspiration	1
Streptococcus equinus	intra-abdominal	1
Streptococcus mitis	blood culture	3
Streptococcus pneumoniae	blood culture	1
	tracheal aspiration	2
Streptococcus spp.	intra-abdominal	1
Streptococcus type C	pleural fluid	1

CSF – cerebrospinal fluid, BAL - bronchoalveolar lavage, PUC - Permanent urinary catheter, MRSA - Methicillin-resistant *Staphylococcus aureus*
